# Nano-encapsulation of essential amino acids: ruminal methane, carbon monoxide, hydrogen sulfide and fermentation

**DOI:** 10.1186/s13568-024-01767-4

**Published:** 2024-09-30

**Authors:** Jorge Adalberto Cayetano De Jesús, Mona Mohamed Mohamed Yasseen Elghandour, Moyosore Joseph Adegbeye, Daniel López Aguirre, José Alejandro Roque-Jimenez, Maximilian Lackner, Abdelfattah Zeidan Mohamed Salem

**Affiliations:** 1 Facultad de Medicina Veterinaria y Zootecnia, Universidad Autónoma del Estado de México, C.P. 50000, Toluca, Estado de México Mexico; 2 Research Centre for Animal Husbandry, National Research and Innovation Agency, Cibinong Science Centre, Cibinong, Bogor 16915, Jl. Raya Jakarta-Bogor Indonesia; 3 Facultad de Ingeniería y Ciencias, Universidad Autónoma de Tamaulipas, 87149 Ciudad Victoria, Tamaulipas, Mexico; 4 Instituto de Ciencias Agrícolas, Universidad Autónoma de Baja California, 21705 Baja California, Mexico; 5 Department of Industrial Engineering, University of Applied Sciences Technikum Wien, Hoechstaedtplatz 6, 1200 Vienna, Austria

**Keywords:** Amino acids, Carbon monoxide, Hydrogen sulfide, Methane, Nano-encapsulation, Rumen fermentation

## Abstract

This study aimed to evaluate the effect of nano-encapsulation of four essential amino acids (AA), threonine, methionine, tryptophan, and lysine on in vitro ruminal total gas, methane, carbon monoxide, and hydrogen sulfide production as well as the rumen fermentation profile in cattle. The highest (*P* < 0.001) rate and asymptotic gas production after 48 h of incubation was observed in the diets that had threonine, followed by lysine, methionine, and tryptophan. Asymptotic methane gas production decreased in the following order: threonine > lysine > tryptophan > methionine (*P* < 0.0001) and the rate of production per hour followed the same trend (*P* = 0.0259). CH_4_ parameters showed that in 4 h, 24 h, and 48 h of incubation the lowest methane production was obtained in the diet with methionine (*P* < 0.05) and the highest one in diet supplemented with threonine. Methane fractions showed that methionine-containing diets resulted in more (*P* < 0.05) metabolizable energy *versus* methane, followed by tryptophan-containing, and then lysine-containing diets. Methionine-fortified diets seem to be the most eco-friendly among those studied regarding methane output. However, based on methane, CO, and H_2_S output as well as the rumen fermentation profile nano-encapsulated lysine is recommended for use in ruminant nutrition.

**Supplementary Information:**

The online version contains supplementary material available at 10.1186/s13568-024-01767-4.

## Introduction

The supply of protein and its amino acid constituents is very important in animal diets, and appropriate amino acid additives can greatly improve animal production (Ren et al. 2019). Adequate amino acid supply for high-producing ruminants is challenging, and to improve amino acid availability to animals it is necessary to protect these nutrients (Albuquerque et al. 2020). The limiting amino acid from plant ingredients and the need to protect amino acids from rumen microbes deamination, especially in high-producing animals necessitates the need to supply exogenous amino acids and protect them with modern techniques to reduce nitrogen excretion. Inefficient use of excess dietary protein brings a metabolic burden and pollutes the environment. Optimizing the use of nutrients has become a top priority due to environmental protection issues (Chen et al. 2021). Therefore, several technologies aiming to protect amino acids from microbial degradation in the rumen were developed (Van den Bossche et al. 2023), to avoid microbial amino acid degradation (Firkins and Mitchell 2023). Various studies reported positive effects on animal performance, nutrient digestibility, and nitrogen metabolism, of supplementary dietary amino acids, in particular methionine and lysine, in ruminant and monogastric diets in their free form as well as in a form for protecting them from microbial degradation (Tsiplakou et al. 2017; Teixeira et al. 2019; Chen et al. 2021). Furthermore, Liu et al. (2021) observed a positive effect of amino acids protected from microbial degradation on the rumen fermentation profile.

However, to the best of our knowledge, no study has considered the effect of amino acid supplementation in ruminant diets on the production of greenhouse gases such as methane and other gases such as CO and H_2_S. Furthermore, no other study has considered the need to add other essential amino acids besides methionine and lysine, for example, threonine and tryptophan leading to little information on their impact on ruminant metabolism and no study has compared these four essential amino acids in the rumen environment. In addition, there is little information on the role of nano-encapsulated amino acids on enteric ruminal methane, H_2_S and the fermentation profile. Thus, this study aims to evaluate the effect of four essential amino acids, namely methionine, lysine, threonine, and tryptophan in their free and nano-encapsulated form on ruminal total gas, methane (CH_4_), carbon monoxide (CO), and hydrogen sulfide (H_2_S) production as well as rumen fermentation profile and CH_4_ conversion efficiency in cattle.

## Materials and methods

### Amino acids

Amino acids (methionine, lysine, threonine and tryptophan) were obtained from Evonik México S.A. de C.V., México. Mesquite gum powder from *Prosopis laevigata* trees was provided by the Universidad Autónoma Metropolitana-Iztapalapa. Gum powder is a highly branched complex polyelectrolyte formed mainly by L-arabinose and D-galactose, and minor proportions of 4-O-methyl-D-glucuronate and L-rhamnose, in a 2:4:1:1 ratio, and a protein content of around 4.8% db (dry basis), which is responsible for its excellent emulsifying properties (Roman-Guerrero et al., 2009). Soybean oil was purchased from a local supermarket (Toluca, State of Mexico, Mexico). Tween 20™ and sodium alginate were acquired from Sigma Aldrich, S.A. de C.V. (Toluca, State of Mexico, Mexico). Distilled water was produced in the lab by reverse osmosis.

### Experimental diet

An experimental diet was formulated to cover the nutritional requirements of cattle in the finishing stage. The composition of the diet was determined in the Bromatology laboratory of the Faculty of Veterinary and Zootechnics of the Universidad Autónoma del Estado de México (Table 1). Representative samples of the experimental diets were taken and dehydrated at 60 °C for 72 h. The dry residues were ground in a hammer mill (Thomas Wiley^®^ Laboratory Mill model 4, Swedesboro, NJ, USA), with a 1 mm sieve. The percentages of moisture, dry matter, ash, nitrogen and ether extract were determined according to the respective AOAC methodologies (1997). The analysis of neutral and acid detergent fiber was performed in an ANKOM200 fiber analyzer (ANKOM Technology Corp, Macedon, NY, USA) with alpha-amylase and sodium sulfite according to Van Soest et al. (1991). The determination of organic matter was calculated by subtracting the ash content. The percentage of protein was determined by multiplying the nitrogen content by 6.25.

**Table 1:** Ingredients and composition of the experimental diet

Ingredients	%
Alfalfa hay	9.1
Wheat grains	25.0
Corn grains	25.0
Bran	13.9
Corn gluten	12.9
Soyabean meal	2.0
Molasses	12.0
Vitamins/Minerals	0.1
*Composition*	
Crude protein (%)	14.73
Ether extract (%)	18.08
Acid detergent fiber	9.49
Neutral detergent fiber	24.67
Free nitrogen extract	66.43
Ca (g/kg)	1.44
P (g/kg)	3.79
Mg (g/kg)	1.71
Na (g/kg)	0.56
K (g/kg)	8.79
Cl (g/kg)	0.68
Zn (g/kg)	22.95
Cu (g/kg)	7.65
Fe (g/kg)	120.14
Digestible crude protein (g/kg SM	108.09

### Preparation of nano encapsulation of the amino acids

#### Preparation of W1/O emulsion

The amino acids were dissolved in distilled water at a 1:1 (w/v) ratio. Tween 20 (6% by weight) was added to the soybean oil at 60 °C and stirred at 800 rpm for 15 min to ensure complete dissolution. Then, the W1/O emulsion (i.e. water, phase 1, is dispersed in oil) was prepared by slowly adding the amino acid solution to the oil phase in a 1:1 (w/w) ratio and mixing using an Ultra-Turrax T50 homogenizer (IKA^®^-WERKE Works Inc., Wilmington, NC, USA) at 8000 rpm for 6 min (Su et al. 2022)

#### Preparation of W1/O/W2 double emulsion

For the external aqueous phase (W2), mesquite gum (10% by weight) and sodium alginate (2% by weight) were dissolved in distilled water. This was then mixed with the freshly prepared W1/O at 25 °C. The W1/O/W2 emulsions were mixed in a (1:2, w/w) ratio with an Ultra-Turrax T50 homogenizer (IKA^®^-WERKE Works Inc., Wilmington, NC, USA) at 8000 rpm for 5 min. The emulsions were stored at 4 °C after preparation (Su et al. 2022).

### Characterization of the emulsion

#### Droplet size measurement

For the measurement of droplet size, the method of Liu et al. (2020) with some modifications was used. The evaluation of the size distribution and the volume-weighted mean diameter of the droplets in the double emulsion was determined using a static light scattering instrument (Mastersizer 3000, Malvern Instruments, Worcestershire, UK).

### Zeta potential measurement

To measure the zeta potential of the droplets, a particle electrophoresis instrument (Zetasizer Nano ZS, Malvern Instruments, Worcestershire, UK) was used (Mikulcova et al. 2018). The samples were diluted 1:100 (w/v) in deionized water under agitation.

### Microstructure of the W1/O/W2 double emulsion

To observe the microstructure of the W1/O/W2 double emulsions, an optical microscope with a 100× oil immersion objective and a confocal laser scanning microscope were used. The W1/O/W2 double emulsions were stained using Nile Red (0.1 mg/mL) and Fluorescein-FITC (0.1 mg/mL), respectively. A 5 µL sample was spread on the microscope slide with a coverslip at 25 °C.

### Encapsulation efficiency

The W1/O/W2 emulsion was mixed with distilled water in a 1:3 (w/w) ratio, then centrifuged at 6500 rpm for 15 min, and the supernatant was collected using a syringe and subsequently filtered using a drain membrane (pore size: 0.45 μm) and stored at 4 °C until analysis.

Amino acid content was determined by high-performance liquid chromatography (Agilent HPLC 1100, USA) using an Agilent Hypersil ODS column (5 μm, 4.0 mm × 250 mm) and a UV detector (λ = 338 nm). The mobile phases consisted of A: 27.6 mmol/L sodium acetate-triethylamine-tetrahydrofuran (500/0.11/2.5, v/v/v) and B: 80.9 mmol/L sodium acetate-methanol-acetonitrile (1/2/2, v/v/v). Elution was performed at a flow rate of 1.0 mL/min with a gradient of 8 to 100% of mobile phase B for 20 min, followed by 100 to 0% of mobile phase B for 4 min. The encapsulation efficiency of the double emulsion was calculated using the following equation:

$${\text{EE}}\;\left( \% \right)=\left( {{\text{M}}0 - {\text{M}}} \right)/{\text{M}}0 \times {\text{1}}00\% $$

Where EE is the encapsulation efficiency (%), M0 is the weight (mg) of the amino acids initially dissolved in the internal aqueous phase (W1) of the W1/O/W2 emulsions, and M is the weight (mg) of the amino acids released to the external aqueous phase (W2) after centrifugation.

### Storage stability of the W1/O/W2 double emulsion

The W1/O/W2 emulsions containing encapsulated lysine, methionine, threonine, and tryptophan were stored at 4 °C after preparation. The external phase was collected every 7 days for a period of 28 days to determine the encapsulation efficiency of the amino acids.

### In vitro ruminal incubation

Ruminal contents were obtained from four slaughtered cattle (350–450 kg live weight) at slaughterhouse in the municipal of Toluca, Mexico, mixed and strained through 4 layers of cheesecloth into a flask with O_2_ free headspace. The rumen contents were transferred in an airtight thermos to the Bromatology laboratory of the Faculty of Medicine, Veterinary and Zootechnics of the Autonomous University of the State of Mexico, Toluca, Mexico. Samples of 500 mg of the diet as a substrate (previously ground and weighed) were weighed into 160 ml serum bottles. After that, 10 ml of particle-free ruminal fluid was added to each bottle, and 40 ml of the buffer solution of Goering and Van Soest (1970), with no trypticase added, was immediately added in a 1:4 (v/v) proportion. The different amino acids (free form, nano-encapsulated) were added to the glass vials in their respective concentrations (lysine: 0, 0.2, 0.4, 0.6 g/g diet DM; methionine: 0, 0.15, 0.3, 0.6 g/g diet DM; threonine: 0, 0.1, 0.15, 0.2 g/g diet DM; tryptophan: 0, 0.08, 0.1, 0.12 g/g diet DM).

A total of 288 bottles (3 bottles of each triplicate sample within each of the 4 amino acids with 4 different levels and in two forms (free and nano- encapsulated), in 3 runs on different weeks, with 3 bottles as blanks (i.e., rumen fluid only), were incubated for 48 h. Once all bottles were filled, they were immediately closed with rubber stoppers, shaken and placed in the incubator at 39 °C. The volume of gas produced, methane, carbon monoxide and hydrogen sulfide production were recorded at 2, 4, 6, 24, 28, 30 and 48 h of inoculation.

At the end of incubation (i.e., 48 h), bottles were uncapped, pH was measured immediately with a pH meter (GLP 22, Crison Instruments, Barcelona, Spain), and fermentation was stopped by swirling the bottles in ice. The contents of each bottle were transferred as filtered fermentation residue to determine the apparently degraded substrate.

### Total gas, methane, carbon monoxide and hydrogen sulfide production

Total gas volume was measured in PSI (pounds per square inch) at 2, 4, 6, 24, 28, 30 and 48 h of incubation as described by Theodorou et al. (1994) using a digital manometer with an accuracy of 2% (Manometer model 407910, Extech^®^ Instruments, Nashue, NH, USA). CH_4_, CO and H_2_S were quantified according to Acosta et al. (2022). At the end of each measurement, the gas accumulated at the top of the vials was released with a syringe without a plunger to avoid further gas accumulation and partial dissolution of the evaluated gases (Tagliapietra et al. 2010).

### Rumen pH and dry matter degradability

After 48 h of incubation, the contents of the glass vials were filtered through 25 mm porosity bags (Filter bag F57, ANKOM Technology Corp., Macedon, NY, USA). The liquor (filtrate) was collected in a glass beaker and the pH was measured immediately using a glass electrode potentiometer (Hanna^®^ Instruments model HALO^®^ HI11102). The residues adhering to the walls of the glass vials were removed by rinsing with distilled water and collected in the same bags as used for the initial filtration. The residues were dried at 60 °C for 72 h. Dry matter digestibility (DMD) was calculated considering the initial weight of the diets and the weight of the obtained dried residues.

### Calculations and statistical analysis

The kinetics of total gas, CH_4_, CO and H_2_S production were estimated by adjusting the gas volume with the NLIN procedure of SAS (2002) according to the model proposed by France et al. (2000):

$${\text{y}}={\text{b}} \times [{\text{1}}-{{\text{e}}^{ - {\text{c}}({\text{t}} - {\text{Lag}})}}]$$

where y = volume (ml) of total gas, CH_4_, CO and H_2_S at time t (h). b = asymptotic production of total gas, CH_4_, CO and H_2_S (ml/g DM). c = production rate of total gas, CH_4_, CO and H_2_S (ml/h). Lag = initial lag time before total gas, CH_4_, CO and H_2_S production starts (h).

Metabolizable energy (ME) (MJ/kg DM) was estimated according to the equation proposed by Menke et al. (1979):

$${\text{ME}}={\text{2}}.{\text{2}}0+\left( {0.{\text{136}} \times {\text{GP}}} \right)+\left( {0.0{\text{57}} \times {\text{CP}}} \right)$$

where CP = crude protein (g/kg DM). GP = total gas production (ml/200 mg DM) at 24 h of incubation.

Total short-chain fatty acid concentrations (SCFA) (mmol/200 mg DM) were calculated according to Getachew et al. (2002) as:

$${\text{SCFA}}=\left( {0.0{\text{222}} \times {\text{GP}}} \right) - 0.00425$$

where GP = total gas production (ml/200 mg MS at 24 h of incubation).

In addition, the ratio of CH_4_ to SCFA (CH_4_: SCFA; mmol mmol-1), ME (CH_4_: ME; g MJ-1) and OM (CH_4_: OM; mL/g) were calculated.

The experimental design was completely randomized with a 4 × 2 × 4 factorial arrangement (4 amino acids – methionine, lysine, threonine, and tryptophan), 2 forms (free and nano-encapsulated), and (4 doses). The data from the three replicates of each treatment were averaged in each run and the averages obtained, for each run, were used as the experimental unit. The analysis was performed using the GLM procedure of SAS (2002) with the following statistical model:

$${{\text{Y}}_{{\text{ijk}}}}=\mu +{{\text{A}}_{\text{i}}}+{\text{ }}{{\text{F}}_{\text{j}}}+{\text{ }}{{\text{D}}_{\text{k}}}+{\text{ }}+{\text{ }}{\left( {{\text{A }} \times {\text{ F }} \times {\text{ D}}} \right)_{{\text{ijk}}}}+{\varepsilon _{{\text{ijk}}}}.$$

where, Y_ijk_ is the response variable, µ is the general mean, A_i_ is the effect of the dietary type of amino acid, F_j_ is the effect of the form of amino acid (free and nano), D_k_ is the effect of extract doses, and (A × F × D)_ijk_ is the effect of the interaction between the type of amino acid, forms (free and nano-encapsulated) and their doses used, and ε_ijk_ is the experimental error. The comparison of means was performed using Tukey’s test, and they were considered significantly different when *p* ≤ 0.05.

## Results

### Total gas production

Regardless of the type and form of the amino acid used in this study, gas production increased throughout the 48-h incubation period (Table 2). Threonine-based diet produced the highest biogas while tryptophan-based diet produced the lowest. In addition, it was observed that gas production was higher in the presence of the free-form of amino acids compared to the respective nano-encapsulated amino acids.

**Fig. 1:**
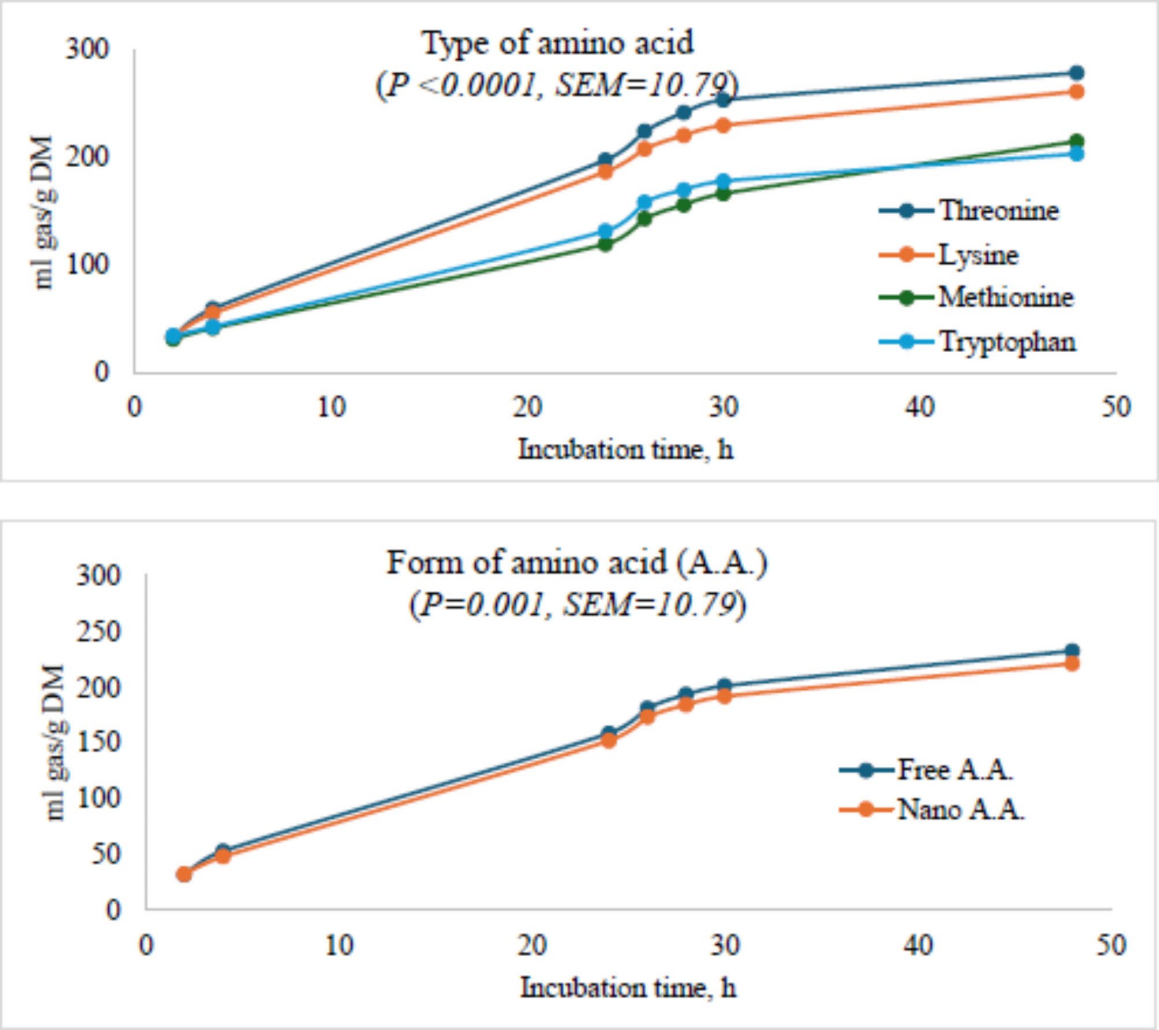
Ruminal total gas production in the presence of threonine, lysine, methionine or tryptophan in their free or nano-encapsulated forms in cattle

The highest asymptotic gas at 48 h and also the highest (*P* < 0.001) rate of production per hour was observed with the threonine-containing diets, followed by lysine-containing, methionine-containing and tryptophan-containing diets. The highest gas production at 4, 24 and 48 h of incubation occured in the presence of threonine followed by lysine while in the presence of methionine, the lowest gas production at 4 h and 24 h was recorded. The form of AA (free vs. nano-encapsulated) did not affect gas production and its kinetics. However, the comparison between free, and nano-form of amino acid showed that 0.2 nano-threonine produced the highest total gas compared to the others. Similarly, 0.1 and 0.12 nano-tryptophan produced the highest total gas compared to other treatments. Furthermore, 0.4 and 0.6 nano-lysine produced more total gas than other doses and the free form of lysine. In contrast to others, 0 ml of free methionine produced higher total biogas than the nano-form of methionine or the other doses under free methionine (Fig. 1; Table 2).

**Table 2:** Ruminal total gas production in the presence of threonine, lysine, methionine or tryptophan in their free forms or nano-encapsulated

Amino acid	Form of AA (FA)	Dose (DA, g/g DM)	Gas production kinetics^a^			Gas production (ml /g DM)		
			b	c	Lag	4 h	24 h	48 h
Threonine	Free	0	259.5	0.031	1.290	68.3	184.7	252.6
		0.1	285.4	0.032	0.876	56.4	191.2	275.8
		0.15	258.6	0.026	2.498	66.3	159.3	244.3
		0.2	323.4	0.030	1.709	58.2	204.2	311.6
	Nano	0	254.8	0.035	0.493	53.1	196.7	251.5
		0.1	291.3	0.034	1.237	59.2	210.5	283.0
		0.15	300.9	0.034	0.790	59.9	215.7	292.7
		0.2	326.1	0.033	1.969	57.3	215.2	315.1
	SEM		24.38	0.0011	0.6910	4.23	17.94	22.90
	FA		0.4351	< 0.0001	0.1875	0.0577	0.0811	0.3103
	DA	Liner	0.2772	0.0211	0.1341	0.4855	0.8648	0.4063
		Quadratic	0.269	0.0808	0.616	0.1769	0.4734	0.2682
	FA x DA		0.6624	0.0407	0.1465	0.0808	0.5835	0.5694
Lysine	Free	0	235.1	0.030	1.468	54.2	164.2	228.6
		0.2	278.1	0.030	1.221	64.4	192.0	269.6
		0.4	256.8	0.030	0.956	53.7	176.8	249.8
		0.6	258.3	0.030	0.808	52.2	176.2	251.1
	Nano	0	248.6	0.029	1.854	59.4	176.9	242.3
		0.2	274.4	0.033	1.014	59.5	199.8	267.6
		0.4	292.1	0.034	1.239	50.1	202.9	284.6
		0.6	291.7	0.032	2.002	50.1	198.2	284.6
	SEM		13.77	0.0014	0.5855	5.71	9.0	13.17
	FA		0.0088	0.008	0.2114	0.604	0.0011	0.0059
	DA	Liner	0.0026	0.0174	0.2211	0.1936	0.0056	0.0023
		Quadratic	0.0368	0.4667	0.5043	0.0263	0.0083	0.0376
	FA x DA		0.1587	0.1189	0.5116	0.505	0.4382	0.1611
Methionine	Free	0	249.0	0.030	0.574	52.2	170.4	241.5
		0.15	243.4	0.030	0.736	48.3	163.1	235.5
		0.3	219.3	0.026	0.735	39.0	122.6	205.7
		0.6	226.6	0.023	0.834	38.6	102.6	202.2
	Nano	0	206.8	0.024	1.120	38.1	107.0	190.4
		0.15	245.5	0.021	1.341	41.0	103.9	211.1
		0.3	258.0	0.021	2.363	37.7	105.0	220.3
		0.6	241.1	0.016	1.952	38.5	88.2	210.1
	SEM		18.72	0.0025	0.7135	3.62	11.69	16.27
	FA		0.7324	< 0.0001	0.0078	0.007	< 0.0001	0.1008
	DA	Liner	0.43	0.0545	0.1351	0.018	0.011	0.7823
		Quadratic	0.3468	0.9463	0.6858	0.2122	0.3493	0.3513
	FA x DA		0.0498	0.7351	0.5999	0.0594	0.0187	0.0256
Tryptophan	Free	0	223.0	0.026	1.534	36.8	114.2	206.2
		0.08	185.2	0.022	2.484	38.4	92.9	167.8
		0.1	178.6	0.025	0.907	34.0	94.8	166.5
		0.12	196.1	0.025	0.596	34.8	98.2	181.7
	Nano	0	208.8	0.028	1.163	40.5	126.6	199.3
		0.08	235.4	0.031	1.706	52.3	166.4	227.9
		0.1	250.4	0.033	1.103	53.5	182.8	243.7
		0.12	230.5	0.030	1.373	50.4	159.2	221.7
	SEM		17.80	0.0015	0.8001	4.08	14.58	17.15
	FA		0.003	< 0.0001	0.9012	0.0002	< 0.0001	0.0006
	DA	Liner	0.8622	0.5911	0.4792	0.3372	0.5801	0.9418
		Quadratic	0.9935	0.1561	0.7096	0.3714	0.2688	0.8161
	FA x DA		0.0466	0.1748	0.4573	0.2586	0.0835	0.0454
SEM			10.79	0.000954	0.404478	2.551043	7.691152	10.04136
P value								
Amino acid (AA)			< 0.0001	< 0.0001	0.5086	< 0.0001	< 0.0001	< 0.0001
Formo of AA (FA)			0.0001	0.0008	0.1266	0.3713	0.0001	0.0002
Dose of AA (DA)			0.0097	0.0072	0.1767	0.0163	0.1918	0.0189
AA x FA x ED			0.2884	0.0266	0.4688	0.0703	0.0839	0.1575

SEM, standard error of the mean

^a^*b* = asymptotic total gas production (ml/g DM); *c* = rate of total gas production (ml/h); *Lag* = initial delay before total gas production begins (h)

### Methane production

**A**ddition of threonine amino acid resulted in the highest volume of methane produced per gram of dry matter, followed by lysine while methionine produced the least. The form of the amino acid used showed that amino acid in free form resulted in the highest methane production as opposed to the nano-encapsulated form. The following amino acid: threonine > lysine > tryptophan > methionine produced asymptotic methane gas in descending order (*P* < 0.0001) and the rate of production per hour followed the same trend (*P* = 0.0259). The diet containing lysine had the quickest time (*P* = 0.0021) for the first volume of methane produced followed by a threonine-containing diet, while the diet containing methionine had the longest delay before methane was produced. CH_4_ production parameters showed that in 4, 24 and 48 h of incubation, diet containing methionine produced the least (*P* < 0.05) methane and it had the lowest proportion of methane for every 100 ml of gas produced while the diet containing threonine did the exact opposite.

**Fig. 2:**
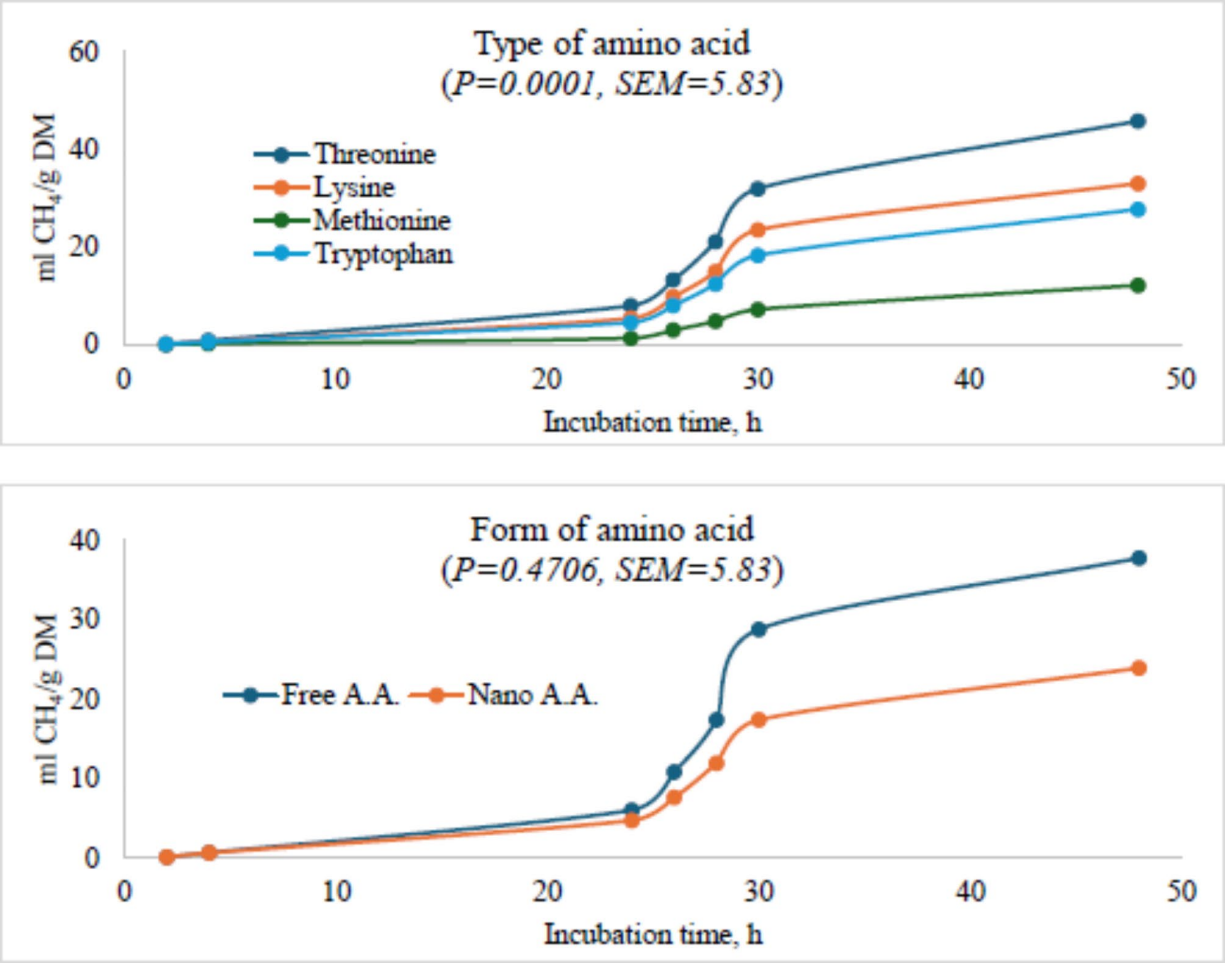
Ruminal methane production in the presence of threonine, lysine, methionine or tryptophan in their free or nano-encapsulated form in cattle

The form of AA did not significantly affect methane production kinetics and CH_4_ production. The interaction of AA, form of AA, and dose showed that there was a difference among treatments (*P* < 0.05). Under threonine, 0.1 free threonine produced the highest methane while 0.15 produced the lowest. However, in the Nano-threonine 0.15 produced the highest methane 0 threonine produced the lowest. Overall threonine performance showed that Nano-threonine led to the production of methane than free-threonine. In the diet containing lysine, 0.6 free lysine produced the lowest methane while the control produced the highest. Inversely, under nano-lysine, the control produced the lowest methane while 0.6-nano-lysine produced the highest. However, the overall trend showed that nano-lysine produced the lower methane which was 3.79-fold lower than the one produced under free-lysine. The general trend for methane production is that free-methionine produced less methane than nano-methionine, which was 2.2-fold lower than that produced by nano-methionine. Under free methionine, all methionine diet produced lower methane compared to the control, with 0.6 methionine producing the lowest. Under nano-methionine, 0.3 and 0.6 methionine produced the highest methane while the control produced the least. Free-tryptophan-containing diet produced the least methane while nano-tryptophan produced the most methane which was 1.82-fold higher. Under free-tryptophan, 0.08 g/g DM produced the highest while 0.1 produced the lowest. Under nano-tryptophan, 0.1 and 0.12 nano-tryptophane produced the highest while the control produced the lowest (Fig. 2; Table 3).

**Table 3:** Ruminal methane production in the presence of threonine, lysine, methionine or tryptophan in their free forms or nano-encapsulated

Amino acid	Form of AA (FA)	Dose (DA, g/g DM)	CH_4_ production kinetics^a^			CH_4_ production (ml /g DM)			CH_4_ (ml100 ml gas)		
			b	c	Lag	4 h	24 h	48 h	4 h	24 h	48 h
Threonine	Free	0	35.6	0.112	3.506	1.11	8.58	38.66	1.63	4.63	15.30
		0.1	22.9	0.118	3.481	0.42	3.75	22.51	0.73	1.96	8.19
		0.15	12.2	0.104	3.047	0.48	2.32	12.02	0.73	1.51	4.97
		0.2	81.4	0.089	2.725	1.58	16.77	80.90	2.70	8.20	25.95
	Nano	0	36.5	0.087	1.770	0.94	7.97	35.82	1.77	4.10	14.27
		0.1	39.0	0.111	2.934	0.59	7.71	38.55	1.00	3.67	13.50
		0.15	90.9	0.118	4.187	1.00	11.11	90.65	1.67	5.17	31.00
		0.2	56.0	0.113	3.582	0.77	8.26	55.88	1.33	3.83	17.45
	SEM		11.0	0.016	0.874	0.271	3.041	11.582	0.427	1.448	3.497
	FA		0.0121	0.8313	0.8752	0.5729	0.5553	0.0172	0.9608	0.876	0.0109
	DA	Liner	0.0926	0.3326	0.1372	0.1331	0.4699	0.1271	0.1049	0.3224	0.2422
		Quadratic	0.1048	0.3437	0.8847	0.0275	0.3477	0.0885	0.0342	0.2495	0.0275
	FA x DA		0.0002	0.1768	0.1246	0.0167	0.0074	0.0002	0.0107	0.0087	< 0.0001
Lysine	Free	0	83.2	0.146	4.581	0.87	10.06	82.38	1.60	6.10	35.77
		0.2	82.1	0.103	3.592	0.96	12.17	81.99	1.50	6.33	30.50
		0.4	30.8	0.090	2.289	0.33	4.53	30.68	0.58	2.47	11.53
		0.6	13.6	0.081	1.368	0.36	3.14	13.53	0.70	1.78	5.39
	Nano	0	12.7	0.082	1.269	0.52	3.10	12.70	0.87	1.76	5.24
		0.2	13.5	0.090	1.546	0.44	3.36	13.51	0.73	1.68	5.04
		0.4	14.2	0.093	2.038	0.37	3.09	14.19	0.73	1.51	4.97
		0.6	14.9	0.083	1.670	0.34	3.30	14.90	0.70	1.66	5.23
	SEM		9.1	0.009	0.864	0.168	1.543	8.910	0.273	0.771	3.189
	FA		< 0.0001	0.0015	0.0046	0.0304	0.0002	< 0.0001	0.0307	< 0.0001	< 0.0001
	DA	Liner	0.0085	0.0037	0.1929	0.0151	0.0416	0.0085	0.0104	0.0105	0.0008
		Quadratic	0.1056	0.2915	0.9605	0.1226	0.0306	0.0975	0.3346	0.0895	0.2059
	FA x DA		0.0011	0.0003	0.0223	0.1172	0.0077	0.001	0.0684	0.0073	0.0003
Methionine	Free	0	12.0	0.089	1.954	0.38	2.56	11.98	0.73	1.51	4.97
		0.15	7.6	0.112	3.505	0.00	1.03	7.63	0.00	0.63	3.23
		0.3	6.1	0.066	3.547	0.00	0.65	5.95	0.00	0.50	2.80
		0.6	5.1	0.087	4.148	0.00	0.46	5.06	0.00	0.45	2.50
	Nano	0	19.7	0.053	5.302	0.19	2.71	18.85	0.47	2.53	10.17
		0.15	35.6	0.109	4.967	0.54	2.48	35.53	1.33	2.47	17.97
		0.3	5.3	0.076	4.789	0.00	0.30	5.17	0.00	0.27	2.33
		0.6	7.2	0.053	4.443	0.00	0.22	6.50	0.00	0.25	3.25
	SEM		8.18	0.024	0.892	0.110	0.959	8.241	0.273	0.870	4.290
	FA		0.1218	0.2194	0.0045	0.3158	0.7325	0.1395	0.2239	0.383	0.1172
	DA	Liner	0.2151	0.994	0.4312	0.0311	0.0462	0.2321	0.0577	0.1005	0.2565
		Quadratic	0.1315	0.0194	0.5676	0.2324	0.8212	0.126	0.1684	0.6754	0.1523
	FA x DA		0.2963	0.4871	0.1963	0.0432	0.8003	0.2952	0.061	0.6502	0.3096
Tryptophan	Free	0	18.4	0.06	5.00	0.17	2.84	18.21	0.47	2.63	10.00
		0.08	36.3	0.12	4.74	0.61	2.87	36.27	1.60	3.10	21.60
		0.1	10.5	0.10	2.94	0.28	1.63	10.52	0.83	1.72	6.35
		0.12	10.7	0.09	2.13	0.37	2.12	10.66	1.08	2.16	5.87
	Nano	0	28.4	0.10	3.12	0.72	5.00	28.33	1.77	4.10	14.27
		0.08	31.2	0.11	3.08	0.52	6.12	31.03	1.00	3.67	13.50
		0.1	40.6	0.10	3.07	0.48	5.69	40.25	0.90	3.12	16.11
		0.12	38.0	0.07	2.08	0.97	7.96	37.70	1.95	4.95	17.20
	SEM		12.13	0.021	1.132	0.225	2.140	12.056	0.428	1.443	5.580
	FA		0.034	0.7872	0.13	0.015	0.0055	0.0347	0.0934	0.074	0.1923
	DA	Liner	0.9256	0.938	0.023	0.1861	0.5225	0.9259	0.2481	0.8745	0.8968
		Quadratic	0.8393	0.2461	0.9034	0.2336	0.581	0.841	0.1296	0.3108	0.8774
	FA x DA		0.2509	0.2038	0.4435	0.1611	0.749	0.2524	0.0419	0.8203	0.1631
SEM			5.84	0.010	0.544	0.112	1.110	5.895	0.202	0.655	2.393
P value											
Amino acid (AA)			0.0001	0.0259	0.0021	< 0.0001	< 0.0001	0.0001	< 0.0001	0.0043	0.0151
Formo of AA (FA)			0.4706	0.2372	0.7317	0.4773	0.4144	0.5118	0.5062	0.9151	0.878
Dose of AA (DA)			< 0.0001	0.0034	0.2362	0.0002	0.0007	< 0.0001	0.0004	0.0035	0.0002
AA x FA x ED			0.0143	0.1785	0.0032	0.2842	0.1226	0.0151	0.0578	0.1682	0.0234

SEM, standard error of the mean

^a^*b* = asymptotic CH_4_ production (ml/g DM); *c* = rate of CH_4_ production (ml/h); *Lag* = initial delay before CH_4_ production begins (h)

### Carbon monoxide production

**Fig. 3:**
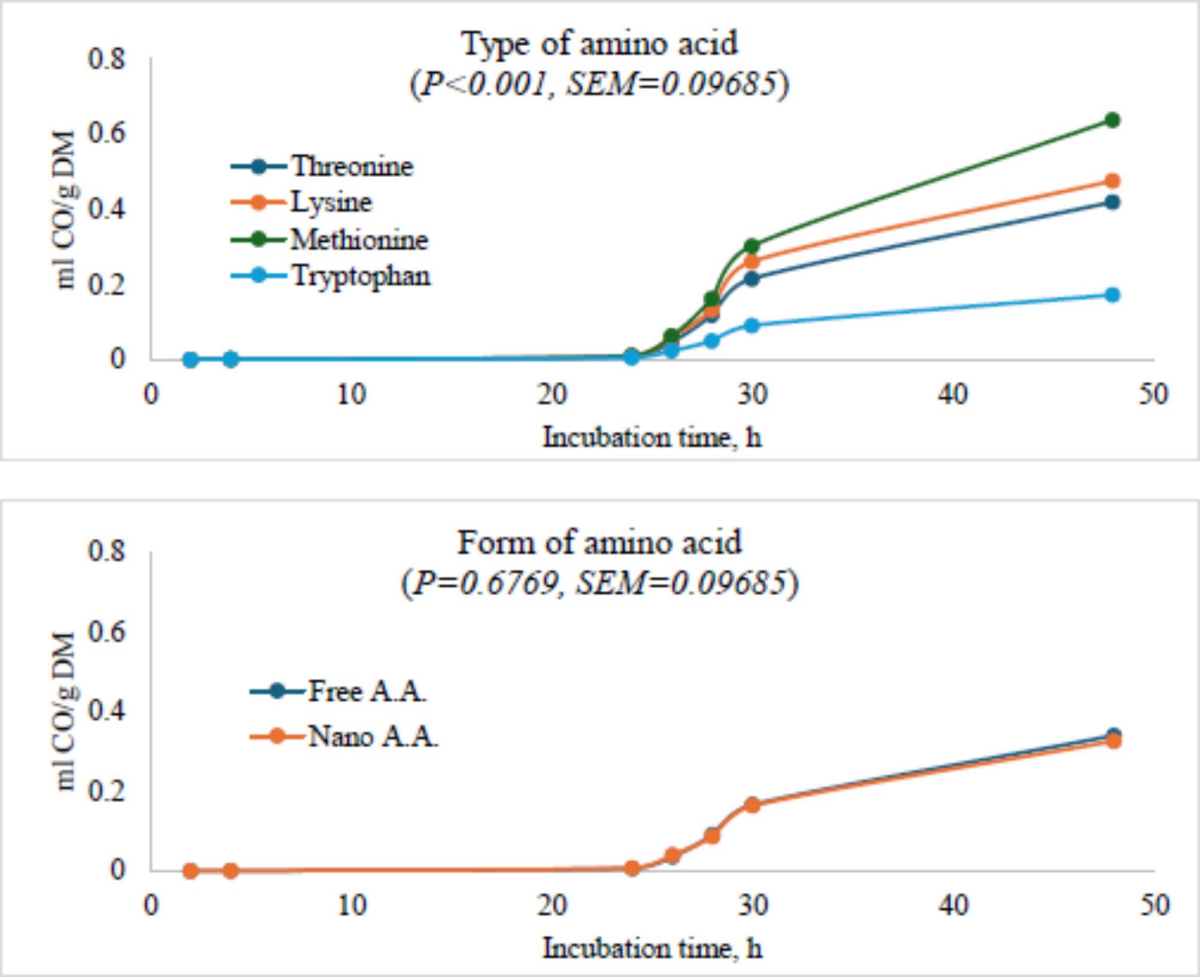
Ruminal carbon monoxide production in the presence of threonine, lysine, methionine or tryptophan in their free or nano-encapsulated forms in cattle

The highest carbon monoxide was produced in the diet containing methionine amino acid while the lowest was recorded in the diet with tryptophan amino acid. The form of amino acid showed that there was no difference between the carbon monoxide produced either the free or nano-encapsulated amino acid. However, the methionine-containing diet had the highest asymptotic CO followed by lysine, and threonine, while the least produced CO was recorded in the tryptophan-based diet. The form of AA did not significantly affect CO, while the dose significantly (*P* < 0.0001) affected CO. Both free and nano-treonine and lysine at 0.2 g/g DM had the highest CO level. In the methionine-based diet, 0.15 g/g DM produced the highest (*P* < 0. 05) CO while the control produced the lowest (*P* < 0. 05). In the free tryptophan-based diet, the highest level of CO was recorded in the control group while 0.12 g/g DM produced the lowest. Nano-tryptophan at 0.10 g/g DM produced the highest (*P* < 0. 05) CO while the control had the lowest (*P* < 0. 05) level of CO. The interaction of amino acid, form of amino acid, and dose of amino acid did not significantly affect the level of CO (Fig. 3; Table 4).

**Table 4:** Ruminal carbon monoxide in the presence of threonine, lysine, methionine or tryptophan in their free forms or nano-encapsulated

Amino acid	Form of AA (FA)	Dose (DA, g/g DM)	CO production kinetics^a^			CO production (ml/g DM)		
			b	c	Lag	4 h	24 h	48 h
Threonine	Free	0	0.4764	0.0006	0.0010	0.0003	0.0044	0.2205
		0.1	0.6645	0.0002	0.0007	0.0002	0.0056	0.3316
		0.15	0.6689	0.0003	0.0009	0.0003	0.0048	0.3077
		0.2	1.0176	0.0005	0.0008	0.0003	0.0074	0.4707
	Nano	0	0.7232	0.0001	0.0086	0.0002	0.0095	0.3437
		0.1	1.4373	0.0015	0.0007	0.0003	0.0108	0.5410
		0.15	1.2185	0.0012	0.0002	0.0004	0.0108	0.5437
		0.2	1.3697	0.0009	0.0024	0.0003	0.0081	0.5578
	SEM		0.10049	0.00036	0.00286	0.00008	0.00131	0.04393
	FA		< 0.0001	0.0154	0.3586	0.682	< 0.0001	< 0.0001
	DA	Liner	0.0002	0.2562	0.1952	0.1369	0.3491	0.0004
		Quadratic	0.0004	0.2675	0.4757	0.3793	0.2966	0.01
	FA x DA		0.012	0.0296	0.5565	0.2306	0.0578	0.1249
Lysine	Free	0	0.8860	0.0004	0.0001	0.0003	0.0073	0.4169
		0.2	1.1592	0.0008	0.0079	0.0006	0.0117	0.5164
		0.4	0.7285	0.0009	0.0045	0.0003	0.0087	0.3537
		0.6	0.7225	0.0013	0.0030	0.0003	0.0086	0.3412
	Nano	0	1.1211	0.0002	0.0015	0.0005	0.0112	0.5218
		0.2	1.2421	0.0002	0.0002	0.0005	0.0144	0.6262
		0.4	1.1424	0.0002	0.0021	0.0002	0.0104	0.5287
		0.6	0.9671	0.0004	0.0006	0.0003	0.0076	0.4538
	SEM		0.18200	0.00047	0.00355	0.00011	0.00157	0.07786
	FA		0.0083	0.039	0.2183	0.9913	0.0278	0.0019
	DA	Liner	0.5549	0.4724	0.4204	0.2309	0.7974	0.5591
		Quadratic	0.031	0.8325	0.4462	0.0087	0.0013	0.0118
	FA x DA		0.5556	0.8644	0.5338	0.3878	0.1978	0.8638
Methionine	Free	0	1.3076	0.0001	0.0014	0.0003	0.0113	0.5524
		0.15	2.7209	0.0034	0.0003	0.0004	0.0311	1.2523
		0.3	2.1719	0.0024	0.0001	0.0002	0.0132	0.9230
		0.6	1.4029	0.0021	0.0002	0.0002	0.0071	0.6755
	Nano	0	0.6171	0.0004	0.0004	0.0002	0.0035	0.2664
		0.15	1.3057	0.0020	0.0007	0.0002	0.0057	0.6046
		0.3	1.1270	0.0013	0.0001	0.0002	0.0062	0.5498
		0.6	0.7834	0.0006	0.0022	0.0002	0.0030	0.3688
	SEM		0.30300	0.00079	0.00084	0.00010	0.00372	0.16464
	FA		< 0.0001	0.0454	0.6374	0.0903	< 0.0001	0.0004
	DA	Liner	0.0081	0.011	0.4184	0.4374	0.4187	0.0203
		Quadratic	0.0024	0.0043	0.9508	0.1948	0.001	0.0052
	FA x DA		0.3222	0.4344	0.5163	0.2225	0.0078	0.4844
Tryptophan	Free	0	0.4258	0.0001	0.0001	0.0001	0.0030	0.1688
		0.08	0.3877	0.0002	0.0009	0.0002	0.0039	0.1413
		0.1	0.2578	0.0001	0.0035	0.0001	0.0027	0.1124
		0.12	0.2560	0.0000	0.0001	0.0002	0.0029	0.1166
	Nano	0	0.3790	0.0000	0.0001	0.0002	0.0054	0.1711
		0.08	0.4272	0.0001	0.0000	0.0003	0.0075	0.1834
		0.1	0.5252	0.0004	0.0001	0.0003	0.0057	0.2473
		0.12	0.4400	0.0001	0.0001	0.0003	0.0060	0.2016
	SEM		0.08472	0.00015	0.00063	0.00006	0.00241	0.03738
	FA		0.0285	0.8402	0.0965	0.0236	0.0341	0.0065
	DA	Liner	0.4235	0.7251	0.9886	0.1406	0.9271	0.7257
		Quadratic	0.775	0.0979	0.0389	0.922	0.9505	0.56
	FA x DA		0.1068	0.3149	0.2055	0.583	0.9902	0.1761
SEM			0.09685	0.00026	0.00114	0.00005	0.00130	0.04679
P value								
Amino acid (AA)			< 0.0001	0.0075	0.8738	< 0.0001	< 0.0001	< 0.0001
Formo of AA (FA)			0.6769	0.0752	0.5343	0.6204	0.5279	0.7281
Dose of AA (DA)			< 0.0001	0.0013	0.9805	0.0083	< 0.0001	< 0.0001
AA x FA x ED			0.1077	0.0728	0.4929	0.313	0.0002	0.1374

SEM, standard error of the mean

^a^*b* = asymptotic CO production (ml//g DM); *c* = rate of CO production (ml/h); *Lag* = initial delay before CO production begins (h)

### Hydrogen sulphide production

**Fig. 4:**
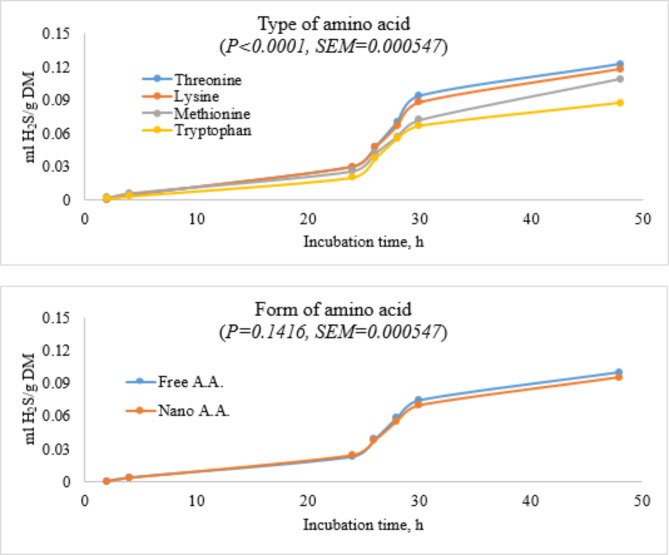
Ruminal hydrogen sulfide production in the presence of threonine, lysine, methionine or tryptophan in their free or nano-encapsulated forms in cattle

Threonine amino acid group had the highest H_2_S while the tryptophan group produced the lowest. The form of amino acids also showed that amino acids in free forms had higher H_2_S than those in nano-forms. Threonine-based diet had the highest (*P* < 0. 05) volume of H_2_S produced in 48 h of incubation, followed by lysine, methionine, and tryptophan. Asymptotic H_2_S showed that a methionine-based diet produced the highest (*P* < 0. 05) level of H_2_S, followed by lysine, then threonine while the tryptophan had the lowest value for asymptotic H_2_S. Nano-tryptophan diet produced more H_2_S than the free-tryptophan-free diet (Fig. 4; Table 5).

**Table 5:** Ruminal hydrogen sulfide in the presence of threonine, lysine, methionine or tryptophan in their free forms or nano-encapsulated

Amino acid	Form of AA (FA)	Dose (DA, g/g DM)	H_2_S production kinetics^a^			H_2_S production (ml/g DM)		
			b	c	Lag	4 h	24 h	48 h
Threonine	Free	0	0.0035	0.0254	0.1013	0.0152	0.1093	0.4224
		0.1	0.0038	0.0276	0.1197	0.0091	0.0665	0.2892
		0.15	0.0061	0.0291	0.1176	0.0232	0.1123	0.4381
		0.2	0.0040	0.0329	0.1377	0.0124	0.1000	0.4290
	Nano	0	0.0032	0.0274	0.1040	0.0101	0.0852	0.3228
		0.1	0.0050	0.0330	0.1287	0.0186	0.1216	0.4674
		0.15	0.0050	0.0340	0.1305	0.0149	0.1013	0.3891
		0.2	0.0037	0.0309	0.1375	0.0118	0.0992	0.4362
	SEM		0.00096	0.00696	0.01586	0.00607	0.04108	0.12102
	FA		0.8036	0.4978	0.4848	0.7117	0.8098	0.877
	DA	Liner	0.0087	0.3297	0.0903	0.1492	0.7313	0.6216
		Quadratic	0.9183	0.7616	0.3081	0.5951	0.7395	0.8371
	FA x DA		0.4717	0.892	0.9469	0.2109	0.5042	0.3782
Lysine	Free	0	0.0050	0.0250	0.0942	0.0125	0.0630	0.2388
		0.2	0.0069	0.0348	0.1193	0.0264	0.1346	0.4585
		0.4	0.0047	0.0289	0.1175	0.0146	0.0941	0.3728
		0.6	0.0036	0.0243	0.1054	0.0109	0.0741	0.3208
	Nano	0	0.0053	0.0311	0.1141	0.0116	0.0674	0.2465
		0.2	0.0052	0.0327	0.1237	0.0199	0.1276	0.4784
		0.4	0.0037	0.0295	0.1317	0.0104	0.0840	0.3750
		0.6	0.0032	0.0301	0.1285	0.0088	0.0871	0.3732
	SEM		0.00106	0.00417	0.01276	0.00441	0.02397	0.07652
	FA		0.1827	0.1945	0.0164	0.1516	0.9944	0.6293
	DA	Liner	0.1965	0.6632	0.021	0.8857	0.1982	0.0388
		Quadratic	0.0395	0.0423	0.3165	0.0012	0.0029	0.0059
	FA x DA		0.5093	0.3815	0.6844	0.8281	0.9171	0.9757
Methionine	Free	0	0.0042	0.0238	0.1139	0.0092	0.0530	0.2516
		0.15	0.0090	0.0418	0.1283	0.0242	0.1128	0.3453
		0.3	0.0072	0.0299	0.1060	0.0153	0.0644	0.2272
		0.6	0.0065	0.0269	0.1100	0.0164	0.0675	0.2741
	Nano	0	0.0027	0.0164	0.0813	0.0076	0.0460	0.2205
		0.15	0.0071	0.0239	0.1067	0.0176	0.0596	0.2666
		0.3	0.0068	0.0254	0.1185	0.0189	0.0698	0.3190
		0.6	0.0059	0.0211	0.1075	0.0200	0.0707	0.3606
	SEM		0.00092	0.00401	0.01281	0.00436	0.01798	0.05026
	FA		0.0239	0.0008	0.1006	0.9215	0.1776	0.6395
	DA	Liner	< 0.0001	0.0229	0.1155	0.0225	0.1842	0.4689
		Quadratic	< 0.0001	0.0032	0.1189	0.0146	0.022	0.2532
	FA x DA		0.5467	0.1401	0.0909	0.4242	0.1171	0.2731
Tryptophan	Free	0	0.0025	0.0173	0.0903	0.0072	0.0502	0.2618
		0.08	0.0037	0.0155	0.0752	0.0083	0.0341	0.1682
		0.1	0.0029	0.0144	0.0677	0.0089	0.0458	0.2040
		0.12	0.0025	0.0131	0.0715	0.0089	0.0450	0.2492
	Nano	0	0.0031	0.0220	0.0822	0.0073	0.0516	0.1916
		0.08	0.0035	0.0254	0.0903	0.0101	0.0740	0.2633
		0.1	0.0040	0.0260	0.0995	0.0143	0.0909	0.3436
		0.12	0.0030	0.0184	0.0847	0.0095	0.0575	0.2638
	SEM		0.00085	0.00495	0.01425	0.00331	0.01800	0.05895
	FA		0.2788	0.0046	0.0846	0.2443	0.0156	0.1353
	DA	Liner	0.9954	0.2711	0.4331	0.4233	0.9806	0.4765
		Quadratic	0.2496	0.3974	0.8743	0.1099	0.1388	0.3674
	FA x DA		0.7593	0.6884	0.2844	0.6637	0.2899	0.0798
SEM			0.00055	0.00290	0.00805	0.00262	0.01460	0.04433
P value								
Amino acid (AA)			< 0.0001	0.007	0.0004	0.1951	0.0498	0.0168
Formo of AA (FA)			0.1416	0.4046	0.0386	0.6974	0.4016	0.1448
Dose of AA (DA)			< 0.0001	0.0336	0.0283	0.0055	0.148	0.0777
AA x FA x ED			0.7542	0.6477	0.5883	0.7525	0.8133	0.9405

SEM, standard error of the mean

^a^*b* = is the asymptotic H_2_S production (ml/g DM); *c* = is the rate of H_2_S production (ml/h); *Lag* = is the initial delay before H_2_S production begins (h)

### Rumen fermentation profile

Lysine-based diet had the lowest (*P* < 0.0001) rumen fluid pH, while the tryptophan-based diet had the highest value. Lysine-based diet yielded the highest SCFA, followed by threonine, and tryptophan and the least SCFA was found in the methionine-based diet. Threonine-based diet produced the highest ME followed by the lysine-based diet, followed by tryptophan, and the least was produced in the methionine-based diet. Methane fractions showed methionine based resulted in more ME being produced compared to methane, followed by the tryptophan-based diet followed by the lysine-based diet while the diet that produced the least ME compared to the methane it produced was threonine and the same pattern was followed for CH_4_:OM and CH_4_:SCFA. The rumen pH of Nano-amino acid is lower (*P* = 0.0367) than free amino acid, but it did not significantly affect other parameters such as DMD, SCFA, ME, CH_4_: ME, CH_4_:OM and CH4:SCFA. The interaction between amino acid and form of amino acid influenced (*P* < 0.05) rumen fluid pH. Nano-threonine and lysine reduced the rumen pH, while the Nano-form of methionine and tryptophan increased rumen pH. The interaction of AA, FA and dose did not significantly affect rumen fermentation profile and CH_4_ conversion efficiency (Table 6).

**Table 6:** Rumen fermentation profile and CH_4_ conversion efficiency in the presence of threonine, lysine, methionine or tryptophan in their free forms or nano-encapsulated

Amino acid	Form of AA (FA)	Dose (DA, g/g DM)	pH	DMD (%)	SCFA mmol/g DM	ME, MJ/kg DM 24 h	CH4: ME (g/MJ)	CH4:OM (ml/g)	CH4: SCFA at 24 h (mmol/mmol)
Threonine	Free	0	6.73	49.59	5.45	7.50	2.25	4.87	21.31
		0.1	6.63	84.59	5.64	7.60	1.35	2.96	10.51
		0.15	6.60	59.77	4.01	6.76	0.66	1.31	7.71
		0.2	6.35	72.02	9.05	9.35	8.33	18.84	53.63
	Nano	0	6.30	64.76	5.80	7.69	2.96	6.53	22.52
		0.1	6.43	55.37	6.21	7.90	3.16	7.25	21.63
		0.15	6.43	68.67	9.56	9.61	5.38	12.49	33.79
		0.2	6.40	69.03	7.27	8.44	2.82	6.64	20.52
	SEM		0.113	15.23	3.77	1.943	2.009	4.573	10.007
	FA		0.0076	0.7715	0.5358	0.5367	0.659	0.5829	0.7953
	DA	Liner	1.000	0.4738	0.6615	0.6611	0.761	0.7008	0.8694
		Quadratic	0.825	0.2806	0.9034	0.9028	0.6358	0.6581	0.3973
	FA x DA		0.0944	0.1417	0.5689	0.5695	0.0144	0.0147	0.0061
Lysine	Free	0	6.63	79.64	7.27	8.44	5.53	11.30	39.92
		0.2	6.03	59.31	8.50	9.07	6.23	13.67	41.43
		0.4	5.70	68.61	7.83	8.73	2.37	5.09	16.17
		0.6	5.80	65.71	7.80	8.71	1.68	3.53	11.67
	Nano	0	6.37	93.05	7.83	8.73	1.65	3.49	11.49
		0.2	5.90	52.72	8.85	9.25	1.69	3.77	10.97
		0.4	5.57	70.12	8.99	9.32	1.54	3.48	9.88
		0.6	5.43	71.39	8.78	9.21	1.66	3.71	10.85
	SEM		0.110	12.962	0.400	0.205	0.770	1.734	5.042
	FA		0.002	0.6064	0.0011	0.0011	0.0001	0.0002	< 0.0001
	DA	Liner	< 0.0001	0.0858	0.0056	0.0056	0.0218	0.0416	0.0105
		Quadratic	0.1976	0.0151	0.0083	0.0083	0.049	0.0306	0.0898
	FA x DA		0.4769	0.7524	0.4383	0.4391	0.0073	0.0077	0.0073
Methionine	Free	0	6.40	90.78	7.55	8.58	1.39	2.88	9.89
		0.15	6.50	74.45	7.22	8.41	0.57	1.16	4.14
		0.3	6.47	93.57	5.42	7.49	0.39	0.73	3.28
		0.6	6.45	80.92	4.53	7.03	0.30	0.52	2.95
	Nano	0	6.33	74.25	4.73	7.14	1.77	3.05	16.60
		0.15	6.57	80.19	4.59	7.06	1.65	2.78	16.17
		0.3	6.53	76.21	4.64	7.09	0.19	0.33	1.75
		0.6	6.60	66.00	3.89	6.71	0.15	0.25	1.64
	SEM		0.118	10.460	0.519	0.267	0.615	1.077	5.704
	FA		0.3309	0.0598	< 0.0001	< 0.0001	0.5651	0.7325	0.3821
	DA	Liner	0.0954	0.7527	0.011	0.011	0.0656	0.0462	0.1007
		Quadratic	0.1444	0.3341	0.3497	0.3495	0.763	0.8212	0.6749
	FA x DA		0.5777	0.3748	0.0187	0.0188	0.75	0.8004	0.6496
Tryptophan	Free	0	6.53	68.91	5.05	7.30	1.85	3.19	17.26
		0.08	6.53	89.39	4.10	6.81	1.96	3.22	20.33
		0.1	6.57	77.47	4.19	6.86	1.11	1.84	11.29
		0.12	6.60	58.97	4.34	6.93	1.42	2.38	14.15
	Nano	0	6.50	85.53	5.60	7.58	3.08	5.62	26.86
		0.08	6.67	69.73	7.37	8.49	3.35	6.88	23.99
		0.1	6.60	58.83	8.10	8.86	2.99	6.39	20.43
		0.12	6.65	65.46	7.05	8.32	4.35	8.95	32.40
	SEM		0.061	12.759	0.648	0.333	1.196	2.404	9.447
	FA		0.1298	0.5346	< 0.0001	< 0.0001	0.0131	0.0055	0.0748
	DA	Liner	0.0182	0.0998	0.58	0.58	0.6651	0.5226	0.8756
		Quadratic	0.7254	0.8328	0.2687	0.2688	0.4512	0.581	0.3102
	FA x DA		0.2601	0.1099	0.0834	0.0836	0.8144	0.749	0.8198
SEM			0.058	7.429	0.773	0.397	0.663	1.414	4.364
P value									
Amino acid (AA)			< 0.0001	0.1689	0.0397	0.0397	0.0265	0.007	0.0397
Formo of AA (FA)			0.0367	0.1461	0.0864	0.0863	0.6158	0.4726	0.9024
Dose of AA (DA)			< 0.0001	0.3858	0.8388	0.8389	0.0079	0.0058	0.0059
AA x FA x ED			0.3467	0.8238	0.3253	0.3253	0.1031	0.0942	0.1303

pH = ruminal pH; DMD = dry matter degradability; SCFA = short-chain fatty acids; ME = the metabolizable energy

CH_4_:SCFA = methane: short-chain fatty acids ratio; CH_4_:ME = methane: metabolizable energy ratio; CH_4_:OM = methane: organic matter ratio

SEM, standard error of the mean

## Discussion

### Gas production

Amino acids (AA) are essential for the optimal performance of ruminants. Most of the dietary AA are extensively catabolized by the ruminal microbes to synthesize AAs and microbial proteins in the presence of sufficient carbohydrates, nitrogen, and sulfur (Gilbreath et al. 2021). Gas production volume during in vitro digestion can be used to predict the potential of a sample to be degraded when fed to a live animal. However, the use of amino acids was to see how important AA as an additive is to animals. The threonine-amino acid group producing the highest gas suggests that threonine supports the quick proliferation of rumen microbes which is even higher than popular amino acids such as methionine and lysine and led to the production of gases. Asymptotic gases are often used as indicators of fermentative activity, although the proportion of gases in the biogas might have different nutritional connotations. The form of amino acids is also important. Amino acids in free form produced more gases than those in nanoform. The reason for this is that nano-amino acid was protected and resisted digestion (Albuquerque et al. 2023). The protected nano-amino acid would be delivered intact to the intestine where it can be maximally absorbed. Meanwhile, the microbes in the free amino acid group will break down the amino acid to ammonia which will prevent it from being passed to the lower part of the gut for absorption in the intestine. In addition, nitrogen excretion increases energy inefficiency in animals, due to the conversion of ammonia to urea in the liver and its reduction allows its use for other productive purposes (Araújo et al. 2019).

Methane (CH₄) is a greenhouse gas, with a global warming potential higher than that of carbon dioxide (CO₂) over a relatively short time frame. The global warming potential of methane is significantly higher than that of carbon dioxide, but its impact diminishes over time. Reducing methane emissions is considered a crucial strategy for mitigating climate change, as it can have a significant impact on the Earth’s radiative balance, especially in the short term. In this study, the diet containing methionine seems to be eco-friendly because it produced the least methane gas and even of the total gas produced, its methane had the lowest proportion of the total gas while threonine-containing diet did the opposite. The possible reason for this is that since diets containing methionine were observed to have the highest carbon monoxide, there was an alteration in metabolic processes that favoured the accumulation of CO instead of CH_4_. Methionine-based diet had the highest level of CO and H_2_S, yet the lowest CH_4_, which suggests that there were limited activities of methanogen or protozoa to ensure their combination for CH_4_ formation. It is surprising in this study that the nano-form of amino acids produced more methane gas than the free-amino acids. However, there seem to be variations among each amino acid, for example, nano-lysine produced lower methane which was lower than the one produced under free-lysine while free-methionine produced less methane than nano-methionine, which was lower than that produced by nano-methionine. Therefore, individual amino acids can influence the effect amino acid form will have on greenhouse gases. The possible reason for high methane in the nano-amino acid group compared to the free-amino acid is that nano-form reduced amino acid available for microbes which reduced the substrates available for them to proliferate which slow down the rate of fermentation. Besides, one of the reasons for increased methane output from ruminants in Africa is low protein quality. Therefore, limiting amino acids caused the accumulation of methane gas. Carbon monoxide is produced by anaerobic microbes as an intermediate metabolic gas during organic matter degradation (Elghandour et al. 2024). Some of the CO dehydrogenase enzymes are capable of reducing carbon dioxide and oxidizing CO by utilizing their catalytic groups for electron transfer and can maintain redox homeostasis during digestion of the feed (Ragsdale 2008). The increase in CO observed in this study can be attributed to the accumulation of the gas as an alternative to its use for methanogenesis.

Dietary sulphur taken by ruminants through feed is converted by rumen microbes and this occurs with amino acids that contain S which ferment them to sulfate (Patra et al. 2017; Elghandour et al. 2014). This sulfate is used together with lactate as a substrate by sulfur-reducing bacteria to produce sulfide, which is combined with H_2_ to form hydrogen sulfide (H_2_S) (Castro et al. 2021). Methionine is a precursor for the synthesis of S-adenosyl methionine, a methyl donor in various methylation reactions. The higher H_2_S observed in a methionine-based diet compared to the other diet may be attributed to its sulfur-containing amino acids nature which donated sulphur for H_2_ resulting in reduced availability for methane formation.

### Rumen fermentation profile

Rumen fermentation profile can be used to assess the influence of diet, substrate, and additive on the digestion, short-chain fatty acid, rumen pH, and digestibility, and metabolizable energy produced during fermentation either in vivo or in vitro. In this study, the rumen pH, may be used to describe the balance between bases and acids of the rumen environment (Laporte-Uribe 2016). Although lysine had the lowest pH, all the pH, in this experiment were within the range optimal for ruminants (Faniyi et al. 2019). Short-chain fatty acid (SCFA) and metabolizable energy (ME) are digestibility and energy value indicators. In this study, lysine-based sample had the highest SCFA which may be attributed to efficiently aided volatile fatty acid formation. This may be attributed to the ability of lysine to improve growth performance and feed efficiency (Yang et al. 2017). However, during gas production, lysine had the second highest gas which means that perhaps other groups were made up of other gases such as CH_4_, CO and H_2_S rather than gas increase related to adequate nutritional fermentation. Methionine had the lowest value for SCFA and ME which suggests that instead of supporting adequate fermentation, it led to the inhibition of methane production and accumulation of other gases such as CO and H_2_S which might have affected the SCFA and ME production. Despite this, the CH_4_ conversion efficiency showed that the methionine-based diet was efficient in producing more ME, SCFA compared to the CH_4_, and better OM digestibility compared to CH_4_ production.

Methionine was the most effective at reducing methane, tryptophan amino acid was most effective at reducing CO and H_2_S production. Methionine based diet produced 1.07% of methane for every 100 ml biogas compared to others like threonine, lysine and tryptophan which produced 4.13%, 2.9%, and 3.18% of 100 ml of biogas, respectively in 24 h. Methionine based diet produced the highest CO at the rate of 0.01 ml per gram DM while other produced less than this. However, based on methane, CO, and H_2_S output as well as the rumen fermentation profile it is recommended to use lysine in its nano-encapsulated form for ruminant nutrition.

## Electronic supplementary material

Below is the link to the electronic supplementary material.

Supplementary Material 1

## Data Availability

Raw data is available from the corresponding author upon reasonable request.
