# Supplementary material for: Nano-encapsulation of essential amino acids: ruminal methane, carbon monoxide, hydrogen sulfide and fermentation
Source: AMB Express. 2024 Sep 30;14:109. doi: 10.1186/s13568-024-01767-4 (PMC11442736; doi:10.1186/s13568-024-01767-4)
Supplement: Supplementary file 1 — Supplementary Material 1 [file 13568_2024_1767_MOESM1_ESM.pdf]

SPRINGER NATURE

Change of authorship request form - Journals (pre-acceptance)

Important information. Please read.

- This form should be used by authors to request any change in authorship (adding/deleting authors) including changes in corresponding authors. This form should not be used for name changes. Please fully complete all sections. Use black ink and block capitals and provide each author's full name with the given name first followed by the family name.

- By signing this declaration, all authors guarantee that the order of the authors are in accordance with their scientific contribution, if applicable as different conventions apply per discipline, and that only authors have been added who made a meaningful contribution to the work.

- Please note, in author collaborations where there is formal agreement for representing the collaboration, it is sufficient for the representative or legal guarantor (usually the corresponding author) to complete and sign the Authorship Change Form on behalf of all authors, **next to the added/removed author(s)**. **(Complete Section 3, followed by Section 6.)**
  In author collaborations where there is no formal agreement for representing the collaboration and **there are more than 10 authors**, one may sign for all, provided the signer appends correspondence that attests that each of the authors have agreed to the change **and the added/removed authors sign the form**. **(Complete Section 3, followed by Section 6.)**

- Please note, we cannot investigate or mediate any authorship disputes. If you are unable to obtain agreement from all authors (including those who you wish to be removed) you must refer the matter to your institution(s) for investigation. Please inform us if you need to do this.

- If you are not able to return a fully completed form within **30 days** of the date that it was sent to the author requesting the change, we may have to withdraw your manuscript. We cannot publish manuscripts where authorship has not been agreed by all authors (including those who have been removed).

- Incomplete forms will be rejected.

- Please return/upload this form, fully completed, to the Journals Editorial Office. The Journal and/or Publisher will consider the information you have provided to decide whether to approve the proposed change in authorship. We may decide to contact your institution for more information or undertake a further investigation, if appropriate, before making a final decision.

SPRINGER NATURE Springer Nature is one of the world's leading global research, educational and professional publishers, created in May 2015 through the combination of Nature Publishing Group, Palgrave Macmillan, Macmillan Education and Springer Science+Business Media.

SPRINGER NATURE

Change of authorship request form - Journals (pre-acceptance)

Section 1: Please provide the current title of manuscript

Manuscript ID no.: AMBE-D-24-00201R2

Title: Deciphering the role of Moringa oleifera seeds and probiotic bacteria on mitigation of biogas production from ruminants

Section 2: Please provide the previous authorship, in the order shown on the manuscript before the changes were introduced. Please indicate the corresponding author by adding (CA) behind the name.

	First name(s)	Family name	ORCID or SCOPUS id, if available
1st author	Jorge Adalberto	Cayetano De Jesús	
nd author	Mona M.M.Y.	Elghandour	
23rd author	Moyosore	Joseph Adegbeye	
4th author	Maximilian	Lackner	
5th author	Abdelfattah Z.M.	Salem*	
th author			
6th author			
87th author			
9th author			
10th author			

Please use an additional sheet if there are more than 10 authors.

SPRINGER NATURE Springer Nature is one of the world's leading global research, educational and professional publishers, created in May 2015 through the combination of Nature Publishing Group, Palgrave Macmillan, Macmillan Education and Springer Science+Business Media.

SPRINGER NATURE

Change of authorship request form - Journals (pre-acceptance)

Section 3: Please provide a justification for change. Please use this section to explain your reasons for changing the authorship of your manuscript, e.g. what necessitated the change in authorship? Please refer to the (journal) policy pages for more information about authorship. Please explain why omitted authors were not originally included and/or why authors were removed on the submitted manuscript.

The mean reason for changing the list of authors was depending on the participation of each one within the experimental, supervision and writing as well as the revision and answering the reviewer's comments. Dr. Daniel López Aguirre and Dr. José Alejandro Roque-Jimenez were revised and edited the manuscript as well as helping with the providing some advises during answering the reviewer's comments. These two new authors were approved their authorship by all the manuscript authors.

Section 4: Proposed new authorship. Please provide your new authorship list in the order you would like it to appear on the manuscript. Please indicate the corresponding author by adding (CA) behind the name. If the Corresponding Author has changed, please indicate the reason under section 3.

	First name(s)	Family name (this name will appear in full on the final publication and will be searchable in various abstract and indexing databases)	Affiliated institute	E-mail address
1st author	Jorge Adalberto	Cayetano De Jesús	Faculty of Veterinary Medicine and Zootechnics, Autonomous University of the State of Mexico, Toluca, Mexico	adal_cay@hotmail.com
2nd author	Mona M.M.Y.	Elghandour	Facultad de Ciencias Agropecuarias y Ambientales, Universidad Autónoma del Estado de Guerrero, México	mmohamede@uaemex.mx
3rd author	Moyosore	Joseph Adegbeye	DDepartment of Animal Production and Health, University of Africa, Toru-Orua, Sagbama, Bayelsa, Nigeria	alanspeco@gmail.com
4th author	Daniel	López Aguirre	Facultad de Ingeniería y Ciencias, Autonomous University of Tamaulipas, Tamaulipas, México	dlaguirre@docentes.uat.edu.mx
5th author	José Alejandro	Roque-Jimenez	Instituto de Ciencias Agrícolas, Universidad Autónoma de Baja California, 21705 Baja California, México	jose.roque@uabc.edu.mx
6th author	Maximilian	Lackner	Department of Industrial Engineering, University of Applied Sciences Technikum Wien, Hoechstaedtplatz 6, 1200 Vienna, Austria	lacknerm@technikum-wien.at
7th author	Abdelfattah Z.M.	Salem*	Faculty of Veterinary Medicine and Zootechnics, Autonomous University of the State of Mexico, 50000 Toluca, Mexico	salem@uaemex.mx
8th author				
9th author				
0 th author				

Please use an additional sheet if there are more than 10 authors.

The mean reason for changing the list

Deli Nazmín Tirado-Gon

SPRINGER NATURE Springer Nature is one of the world's leading global research, educational and professional publishers, created in May 2015 through the combination of Nature Publishing Group, Palgrave Macmillan, Macmillan Education and Springer Science+Business Media.

SPRINGER NATURE

Change of authorship request form - Journals (pre-acceptance)

Section 5: Author contribution, Acknowledgement and Disclosures. Please use this section to provide a new disclosure statement and, if appropriate, acknowledge any contributors who have been removed as authors and ensure you state what contribution any new authors made (if applicable per the journal or book (series) policy). Please ensure these are updated in your manuscript - after approval of the change(s) - as our production department will not transfer the information in this form to your manuscript.

New acknowledgements:

New Disclosures (financial and non-financial interests, funding):

New Author Contributions statement (if applicable per the journal policy):

State 'Not applicable' if there are no new authors.

SPRINGER NATURE Springer Nature is one of the world's leading global research, educational and professional publishers, created in May 2015 through the combination of Nature Publishing Group, Palgrave Macmillan, Macmillan Education and Springer Science+Business Media.

SPRINGER NATURE Change of authorship request form - Journals (pre-acceptance)

Section 6: Declaration of agreement. *All* authors, unchanged, new and removed *must* sign this declaration.

(NB: Please print the form, (docu)-sign and return/upload a scanned copy. Please note that signatures that have been inserted as an image file are acceptable as long as it is handwritten. Typed names in the signature box are unacceptable.) *** Please delete as appropriate. Delete all of the bold if you were on the original authorship list and are remaining as an author.**

	First name	Family name		Signature	Date
1st author	Jorge Adalberto	Cayetano De Jesús	I agree to the proposed new authorship shown in section 4 ~~/and the addition/removal*of my name to the authorship list~~ /and the proposed change in corresponding author		16-08-2024
2nd author	Mona M.M.Y	Elghandour	I agree to the proposed new authorship shown in section 4 ~~**/and the addition/removal*of my name to the authorship list**~~ /and the proposed change in corresponding author		16-08-2024
3rd author	Moyosor	Joseph Adegbeye	I agree to the proposed new authorship shown in section 4 ~~**/and the addition/removal*of my name to the authorship list**~~ /and the proposed change in corresponding author		16-08-2024
4th authors	Daniel	López Aguirre	I agree to the proposed new authorship shown in section 4 ~~/~~**and the addition~~/removal*~~of my name to the authorship list** ~~/~~and the proposed change in corresponding author		16-08-2024
5th author	José Alejandro	Roque-Jimenez	I agree to the proposed new authorship shown in section 4 /**and the addition/~~removal*~~of my name to the authorship list** /and the proposed change in corresponding author		16-08-2024
6th author	Maximilian	Lackner*	I agree to the proposed new authorshi[illegible] in section 4 ~~**/and the addition/remova**~~[illegible] ~~**name to the authorship list**~~ ~~/~~and the p[illegible] change in corresponding author	Signiert von: Maximilian Lackner<br>Datum: 16.08.2024 21:08:41<br>TRUST<br>Dieses Dokument ist digital signiert!<br>Dieses mit einer qualifizierten elektronischen Signatur versehene Dokument hat gemäß Art. 25 Abs. 2 der Verordnung (EU) Nr 910/2014 vom 23. Juli 2014 ("eIDAS-VO") die gleiche Rechtswirkung wie ein handschriftlich unterschriebenes Dokument.<br>Prüfinformation: Informationen zur Prüfung der elektronischen Signatur finden Sie unter: www.a-trust.at/pdf	16-08-2024
7th author	Abdelfattah Z.M.	Salem	I agree to the proposed new authorshi[illegible] in section 4 ~~**/and the addition/remova**~~[illegible] ~~**name to the authorship list /**~~and the proposed change in corresponding author		16-08-2024

SPRINGER NATURE Springer Nature is one of the world's leading global research, educational and professional publishers, created in May 2015 through the combination of Nature Publishing Group, Palgrave Macmillan, Macmillan Education and Springer Science+Business Media.

SPRINGER NATURE

Change of authorship request form - Journals (pre-acceptance)

	First name	Family name		Signature	Date
8th author			I agree to the proposed new authorship shown in section 4 ~~**/and the addition/removal*of my name to the authorship list** /~~and the proposed change in corresponding author		
9th author			I agree to the proposed new authorship shown in section 4 ~~**/and the addition/removal*of my name to the authorship list**~~ /and the proposed change in corresponding author		
10th author			I agree to the proposed new authorship shown in section 4 /**and the addition/removal*of my name to the authorship list** /and the proposed change in corresponding author		

Please use an additional sheet if there are more than 10 authors.

In case of author collaborations with formal agreement:

	Name of consortium/consortia	First name	Family name		Signature	Date
Representative/legal guarantor				I agree to the proposed new authorship shown in section 4 /**and the addition/removal*of my name to the authorship list** /and the proposed change in corresponding author		

Both added/removed authors should complete the information in the first table under Section 6.

---- End of form ----

SPRINGER NATURE Springer Nature is one of the world's leading global research, educational and professional publishers, created in May 2015 through the combination of Nature Publishing Group, Palgrave Macmillan, Macmillan Education and Springer Science+Business Media.
